# ALGOS: the development of a randomized controlled trial testing a case management algorithm designed to reduce suicide risk among suicide attempters

**DOI:** 10.1186/1471-244X-11-1

**Published:** 2011-01-02

**Authors:** Guillaume Vaiva, Michel Walter, Abeer S Al Arab, Philippe Courtet, Frank Bellivier, Anne Laure Demarty, Stephane Duhem, Francois Ducrocq, Patrick Goldstein, Christian Libersa

**Affiliations:** 1 Département Universitaire de Psychiatrie & Pole de l'Urgence, Lille University Hospital, Lille, France; 2 Clinical Investigation Center 9301, INSERM et CHU Lille, Lille, France; 3 Brest University Hospital & JE 2535, UBO, Brest, France; 4 INSERM U888, Montpellier University Hospital, Montpellier, France; 5 INSERM U797, Pole de Psychiatrie, CHU de Créteil, Hôpital Henri Mondor & Paris 12 University, Créteil, France; 6 SAMU 59 et Pôle de l'Urgence, Lille University Hospital, Lille, France

## Abstract

**Background:**

Suicide attempts (SA) constitute a serious clinical problem. People who attempt suicide are at high risk of further repetition. However, no interventions have been shown to be effective in reducing repetition in this group of patients.

**Methods/Design:**

Multicentre randomized controlled trial.

We examine the effectiveness of «ALGOS algorithm»: an intervention based in a decisional tree of contact type which aims at reducing the incidence of repeated suicide attempt during 6 months. This algorithm of case management comprises the two strategies of intervention that showed a significant reduction in the number of SA repeaters: systematic telephone contact (ineffective in first-attempters) and «Crisis card» (effective only in first-attempters). Participants who are lost from contact and those refusing healthcare, can then benefit from «short letters» or «postcards».

**Discussion:**

ALGOS algorithm is easily reproducible and inexpensive intervention that will supply the guidelines for assessment and management of a population sometimes in difficulties with healthcare compliance. Furthermore, it will target some of these subgroups of patients by providing specific interventions for optimizing the benefits of case management strategy.

**Trial Registration:**

The study was registered with the ClinicalTrials.gov Registry; number: NCT01123174.

## Background

Suicidal behaviors are multifactorial phenomena. It is therefore difficult to define only one strategy to manage a suicidal attempt (SA) for the whole population [1]. In a review of literature of the last 25 years, considering only randomized controlled trials (RCT) with suicidal repetition as primary endpoint, we only find 7 positive trials out of 24 studies. However, all studies show an improvement of compliance to the healthcare plan, which has led some to say that it doesn't matter what is suggested to suicide attempters, as long as they are offered something [2,3].

The seven positive trials can be divided into two categories: intensive intervention programs (nursing at home [4], and a series of Brief Psychotherapy Interventions (IPB) [5]) and, case management programs.

The strategies of intensive intervention demonstrate their effectiveness in reducing the number of SA repetitions at short and medium term, but their weakness lies in the institutional heaviness of deployment and their expensive financial costs.

The other case management strategies have one common point: they are distinct from classical interventions, by proposing a "stay in contact" program, which does not invade the daily life of suicidal attempter, and can be employed in parallel to the eventual healthcare and offers a reliable and effective treatment in cases of suicidal crisis. This kind of case management has inspired the pioneer Jerome Motto about the neologism of "connectedness" [6].

Each one of these strategies is of a great interest in certain categories of suicide attempters. They differ from the first two ones by having not only a lower financial cost but also an easier set up for the entire population in a given territory.

In the "SYSCALL" study, 605 suicidal patients discharged directly from Emergency Departments (ED) were included [7]. A specially trained psychologist contacted patients by telephone, one or three months after the SA. The aim of this psychological supportive intervention was to evaluate the success of the treatment plan defined during the ED stay and eventually adjust it. Over the 13 months follow-up, there were 150 participants who reattempted suicide, 48 of them before the one month' telephone contact. Considering only the subjects effectively contacted (per protocol analysis), the telephone contact at one month proved to be very effective, by reducing to about the half the number of suicidal reattempts over one year (12% versus 21.6% in the control group). The post-hoc analysis showed no effect of the contact in first-attempters. The proposal of telephone contact was very well accepted and positively perceived by the population of this study. In addition, no major side effects were reported by the participants [8].

The strategy based on the delivery of a "crisis card" was proposed by the English team of Bristol and was especially interesting for first-attempters. In addition to usual treatment, the intervention group was offered a "resource card" with the telephone number of a junior doctor in psychiatry available 24 hours a day. The intervention had a significant effect on the rate of SA repetition at 6 months in the first attempters subgroup only, compared to a control group (odds ratio 0.64, 95% CI 0.34-1.26) [9]. The beneficial effect observed at 6 months was not maintained at one year [10].

Jerome Motto proposed the strategy of sending letters to maintain contact with patients at high risk of suicide, who refused to remain in the healthcare system. Patients were contacted by short letters, sent by a person who met them during their hospital stay. The letters were personalized whenever possible. A self-addressed, unstamped envelope was always enclosed. These letters were sent monthly for four months, then every two months for eight months, and finally every three months for four years (24 letters in total). The objective was to make the patient realize that there is a person concerned about him (its existence), and who maintains positive feelings towards him, hence the neologism of "connectedness" proposed by the author. This study included 3.005 patients admitted to hospital for a depression or a suicidal crisis in San Francisco from 1969 to 1974. Thirty days after hospital discharge, subjects were questioned by telephone about adherence to the defined therapy plan; non-compliant subjects were then randomized into two parallel groups with (N = 389) or without sending letters (N = 454). The primary endpoint of the study was to evaluate the impact on suicide rate. This study showed contrasted results at 5 and 15 years. Patients in the contact group had a lower suicide rate at five years (15/389 vs 21/454). Formal survival analyses revealed a significantly lower rate in the contact group (p = .04) for the first two years; differences in the rates gradually diminished, and by year 14 no differences between groups were observed (25/389 vs 26/454) [11].

Like Motto's intervention, an Australian study tested the effectiveness of a programmed systematic sending of a postcard (postcards from the EDge project) during the year following the SA [12]. The intervention consisted of a postcard sent to participants in a sealed envelope at 1, 2, 3, 4, 6, 8, 10, and 12 months after discharge (a total of 8 postcards). The message was the same for all postcards: «*It has been a short time since you were here at the hospital, and we hope things are going well for you... if you wish to drop us a note, we would be happy to hear from you»*. The evaluation concerned all deliberate self poisoning patients admitted for few days in a toxicology unit. The initial follow-up was for 12 months, completed later with a one-year extension [13]. The proportion of SA repeaters in the intervention group did not differ significantly from that in the control group (15.1% vs. 17.3% at one year, 21.2% vs. 22.8% at two years). However, among SA repeaters there was a lower number of reattempts in contact group (incidence risk ratio 0.55 at one year, and 0.49 at two years).

These findings were recently replicated by the New Zealand team of Beautrais: 327 suicide attempters aged 16 years or older, presenting consecutively to ED were included [14]. The intervention consisted of sending four "postcards" to participants at two weeks, 1, 3 and 6 months. Patients allocated to control group did not receive any postcards. All subjects received a standard treatment in parallel. The number of SA repeaters was significantly lower in intervention group (31/153, 20.3%) than in control group (88/174, 50.6%).

Thus, by taking into consideration the strengths and limitations of each of these four strategies, we propose to construct a decisional tree of contact type, a case management algorithm. This monitoring algorithm entitled «ALGOS» is based on the two interventions that showed a significant reduction in the number of SA repeaters: systematic telephone contact (ineffective in first-attempters) and «crisis card» (effective only in first-attempters). Participants non contacted during phone call periods and those refusing proposed healthcare, can then benefit from the «short letters» of Motto or the «postcards» of Carter.

## Aims and Hypothesis

The hypothesis is that deployment of «ALGOS» algorithm following a SA, in parallel to usual treatment; will contribute to the reduction of suicidal behaviours at the six months follow-up. ALGOS algorithm would be more a «crisis management plan», rather than a «case management plan».

The main objective is to evaluate the effectiveness of the algorithm in reducing the number of SA repeaters during a six months period, compared to a control group of suicide attempters benefiting from usual treatment.

Secondary objectives to be studied are:

- Evaluating, according to the method validated by Beecham in 1992 [15], the medical and economic impact within the year following the introduction of ALGOS algorithm

- Evaluating the reduction of other suicidal behaviours at 6 months (decrease of the total number of suicide re-attempts in each group, evolution of Beck's suicidal ideation score, etc ...)

- Assessing the maintenance of the algorithm is effectiveness on suicidal behaviour at 13 months

- Evaluating the differences in the delay of SA repetition within the two groups during the deployment of the algorithm at 6 months and 13 months

- Finally, a more qualitative assessment may propose different responding profiles according to the psychopathology spotted by MINI, the character of being first-attempters or not, sex, etc...

## Methods/Design

It is a multicentre, prospective, comparative, single-blind, randomized controlled trial (ClinicalTrials.gov; number: NCT01123174). It was authorized by AFSSAPS (French Health Ministry) and approved by the Northwest IV Ethical Committee for the Protection of Persons.

## Setting

23 national centres employing psychiatrists and emergency physicians, who are strongly implicated in the treatment of suicidal patients and benefiting from a close collaboration, participate in this program: CHU Angers, CHU Brest, CHU Caen, CHU Clermont Ferrand, CHU Créteil Henri Mondor, CHU Lille, CHU Marseille, CHU Montpellier, CHU Nancy, CHU Nantes, CHU Nice, CHU Paris HEGP, CHU Rennes, CHU Toulouse, CH Boulogne, CH Douai, CH Dunkerque, Polyclinics of Henin Beaumont, CH of Montauban, CH Quimper, CH Roubaix, CH Tourcoing, CH Vannes.

The deployment of the algorithm is supervised by the University Hospital of Lille and the evaluations are carried out by the Clinical Investigation Centre (CIC 9301). The research team has been practising these procedures of follow-up for more than 10 years (these telephone contacts), were realized as a part of previous studies, such as SA patients and road traffic accidents victims. These telephonic assessments are conducted by specially trained psychologists using validated landmarks.

## Participants

Inclusions can be done during the ED stay or at the discharge from hospital, within 7 days after SA. 900 subjects, men or women aged over 18 years, and surviving a SA, whatever the mode of SA is, will be included. Patients with 4 or more SA during the past three years (multi repeaters) will not be included.

The participants will give their free, informed, dated and signed consent.

## Randomisation

Once the patient is included, the investigator randomizes him into one of two groups, intervention (ALGOS) group or control group, using randomization envelopes. Hence the trial is single-blind, the investigator knows the allocated group but not the patient.

Randomization done by blocks is centralized at the CIC of Lille, who follows the inclusions and their distribution in each group. If asymmetrical distribution appears after an intermediate observation of the first 450 inclusions, it will be possible to make rebalance between groups, by a person exterior to the study, according to the number of SA.

## Procedure

After verification of inclusion criteria, a physician informs and answers any questions of the participant in order to get his «consent for a following contact». The participant is then randomized and allocated to ALGOS group or control group. The physician collects the following data: socio-demographic characteristics (including place of birth and place of residence), number of previous SA and headlines of the "care plan defined in ED" (presence or absence of a companion at hospital discharge, recommended psychiatric care, scheduled appointments, prescription of psychotropic drugs, etc ...).

## Intervention

### For ALGOS patients group

#### 1. Delivery of a «Crisis Card»

**Figure 1:**
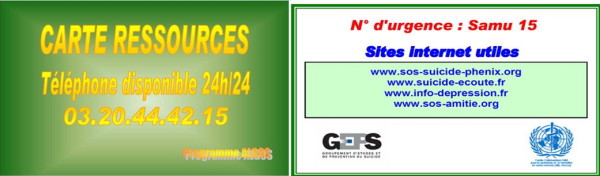
**Example of crisis card**.

All participants surviving a first SA receive a «crisis card» with the emergency phone numbers of the centre where they are included. This card is inspired from the proposed "crisis card" of Evans'team: format of a credit card, green (the colour of hope), plastified, identical for the whole territory containing the logos of suicide prevention organizations (with websites addresses) and personalized with the phone number of the 24 h care permanence of the concerned centre (see Figure 1) [10].

#### 2. Telephone contact

As regards the other patients (not first-attempters), a phone call is conducted between the 10th and 21st day after the SA. The trained psychologist ensures as possible the identity of the patient and gets only addressed to the patient himself. The phone call is done on behalf of the initial unit where the patient was included. The telephone contact presents a part of a psychological support, which is based mainly on empathy, reassurance, explanation and suggestion. The aim of this interview is to verify the adequacy of responses to the existing healthcare, and otherwise to encourage and advise the patient to make new contacts. Three types of phone calls are distinguished according to the time of collecting responses [7]:

- Ordinary call (the most common): the treatment plan is still actual and valid, or the crisis situation that the participant lived improves. In this case, no help or advice seems necessary.

- Participant in difficulty or non compliant: the treatment plan is no longer valid or not followed, unwillingness exists, or the subject is in psychological difficulty: a new treatment plan is eventually suggested, which is already tried during the phone call. In addition, the concerned contact centre will send "postcards" during the following five months.

- Identification of participant at high risk of suicide: the patient is still victim of intrusive suicidal thoughts, asking or not for help. The investigator asks the patient to go to the ED where he was originally treated, to be received by a doctor informed by the concerned contact centre. In case of refusal by the participant, his general practitioner or the mobile emergency medical teams "SAMU/Centre 15" are called. The concerned contact centre will send "postcards" during the following five months.

Telephone contact will be abandoned, if unsuccessful after at least 3 call attempts on 3 different days at 3 different daytimes, and sending "postcards" will be scheduled for the next 5 months.

In all cases, a report of the telephone calls (or if the participant is lost of contact, the information that we have failed to call him) is sent to the general practitioner and eventually to the psychiatrist treating patient.

#### 3. Postcard sending

**Figure 2:**
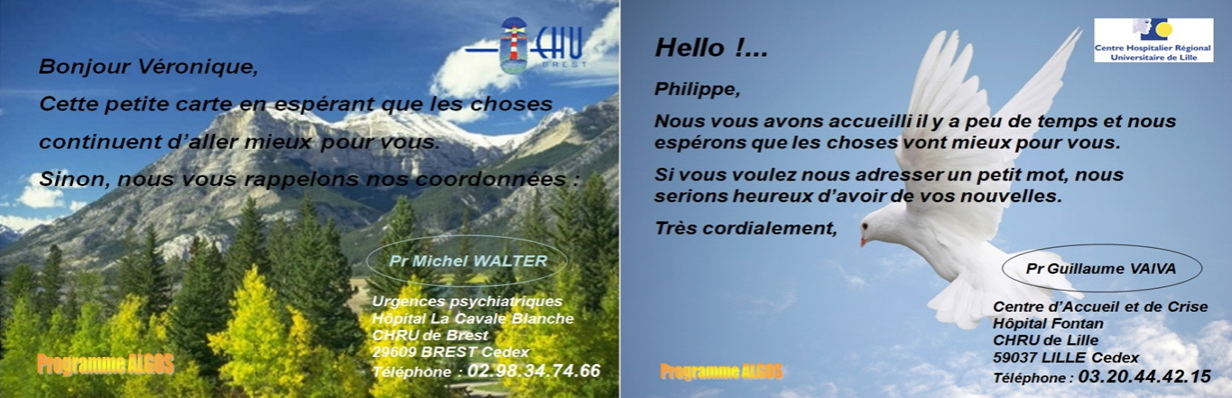
**Examples of postcards**.

This intervention will consist of a programmed sending of postcards at M2, M3, M4 & M5 (see Figure 2) to:

• Participants candidate for telephone contact but not available.

• Participants contacted by telephone but refusing further care, or non-compliant.

• Participants identified during the phone call as in difficulty or experiencing suicidal crisis.

These cards are sent in sealed envelopes. They have identical background (but different forms depending on the month) for all participants in the study. Furthermore, they are personalized with the name of suicide attempter, the signature of the physician who included the patient, and the logo of the hospital where the patient was originally treated. The cards include the phone numbers of the care permanence.

In case of the patient wishes to reply (return letter for the investigator), a new telephone contact will be attempted.

Écouter

Lire phonétiquement

### Control group = process as usual

**Figure 3:**
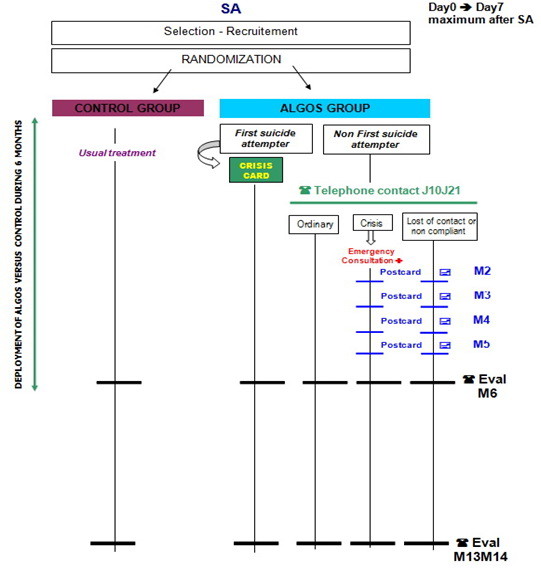
**Study design**.

The participants randomized into the control group do not benefit from any specific treatment and receive the usual care, which is in most cases referral back to their general practitioner (see Figure 3).

## Benefits and Risks

We aim at reducing the number of suicide repeaters in ALGOS group. The telephone contact at both 6 and 13 months will allow the reorientation of all the participants still in difficulty to a suitable healthcare plan, whatever the treatment group they were in.

The evaluators are well trained to assess suicidal risk. If a suicidal risk is detected, the participant will be rapidly managed, by the ED which he belongs to within a maximum of 24 hours.

A possible risk is the potential psychological impact of the interventions planned in the algorithm program. The most invasive procedure of the algorithm (phone call 15 days after SA) demonstrated its safety and its good support by the contacted patient [8].

## Research for Loss of Contact

In this kind of study, the data may be biased by an excessive number of patients included and lost of contact. To limit this bias and improve the data quality, the participants remained lost of contact for the final evaluation, will be sought for by contacting their general practitioner as well as by the compilation of active files and records of ED involved. For participants completely lost of contact, postal requests will be sent to the mayors of their birthplace and home town for the status "alive or not". In case of notification of death (whatever the reason), and after the declaration of a serious adverse event, a member of the research team will question the general practitioner in order to qualify a suicide or not.

## Outcomes

The primary endpoint is the number of participants who reattempted suicide in each group at 6 months.

The secondary endpoint is the total number of deaths by suicide in each group at 6 months. The psychopathological assessment is done through a semi-structured interview of MINI to observe the presence or absence of any psychiatric disorder according to DSM IV at 6 months [16]. Suicidal ideation is assessed by Beck' suicide ideation score in each group at 6 months [17].

The medico-economic assessment is carried out by the method validated by Beecham & Knapp at 6 months [15]. The healthcare contacts are also evaluated in order to compare the cost of ALGOS strategy to those of potentially prevented suicidal behaviours.

To assess the effect of ALGOS algorithm on the long term outcome, the same measurements will be realized at 13 months.

Écout

## Sample Size Calculation

The aim of the study is to compare the repetition rate of SA according to two different modes of intervention at 6 months.

In SYSCALL study, we observed a difference in the repetition rate of about 10% over 12 months, between the group contacted at 1 month and the control group. In the same study, the repetition rate at 6 months was 17.6% in the group with usual treatment and 9.6% in the intervention group [7].

If we consider the repetition rate of SA at 6 months as an endpoint, a rate of 17.6% in group B (CONTROL) and 9.6% in group A (ALGOS), 409 participants per group are required for a 90% statistic power. With a rate of loss of contact estimated to about 10% (in the previous study SYSCALL, we observed 9.2% loss of contact), 450 participants per group are to be included.

Lire phonétiquement

Dictionnaire -

Écouter

Lire phonétiquement

Dictionnaire -

1. **verbe**

1. consider

2. envisage

3. contemplate

4. vision

## Statistical method

The following statistical analysis will be performed:

- Control and descriptive analysis of data: numerical parameters will be summarized by their mean, standard deviation and median. The frequencies percentage will be provided with their confidence interval at 95%.

- Comparisons of means will be realized using Student test (*t *test) or analysis of variance for comparisons according to several factors. In case of multiple comparisons, Bonferroni correction will be applied. Comparisons of frequency percentage will be performed using the Chi square test or exact Fischer if necessary.

- Analysis of predictive factors of SA repetition will be performed using logistic regressions. An approach by a decisional tree (CHAID) will also be considered.

- Analysis of time delay of SA repetition will be performed using conventional methods of survival analysis: Kaplan-Meyer method, log-rank test and Cox model for multifactorial models.

- The research for specific profiles will be conducted using classification methods to identify clusters with atypical profiles.

- The concomitant drug treatments, particularly psychotropic drugs will be taken into consideration in subsequent analysis to ensure that they do not induce bias in the results.

## Discussion

ALGOS algorithm tries to integrate the limits identified in controlled trials of "case management" in which the effectiveness was limited to some specific subgroups of suicide attempters. Based on the published results in this field, the aim of ALGOS study is to propose an algorithm of case management (monitoring) that will target some of these subgroups of patients by providing them specific interventions and thus optimizing the benefits of this strategy.

Most of the centres participating in ALGOS study have skilled expert teams in suicide attempters' care and have sometimes developed specific interventions that differ from one centre to another. This variability of the current care in the "control" group will make the effectiveness of ALGOS algorithm more powerful if demonstrated.

However, while other studies tried to study the effects of interventions on long-term, the impact of ALGOS algorithm will be evaluated at short and medium term (6 and 13 months after SA). If the effectiveness of the algorithm is observed, future researches will focus on assessment at longer periods knowing that the effect of these types of intervention seems to fade away with time.

Finally, the studies showing an effectiveness on reducing SA repetitions often employed heavy and expensive interventions in terms of human and financial resources [4,5]. If the effectiveness of ALGOS algorithm is demonstrated, this easily reproducible and inexpensive strategy will allow supplementary (or alternative) perspectives to the usual therapeutic care for a population sometimes in difficulty with healthcare compliance. In this context, we will evaluate the medico-economic impact of ALGOS algorithm and thus its possible generalization in terms of public health.

## Competing interests

The authors (GV, ASaA, MW, PC, FB, ALD, SD, FD, PG, CL) declare that they have no competing interests:

- In the past 5 years, they didn't received reimbursements, fees, funding, or salary from an organization that may in any way gain or lose financially from the publication of this manuscript.

- They doesn't hold any stocks or shares in an organization that may in any way gain or lose financially from the publication of this manuscript, either now or in the future.

- They don't received reimbursements, fees, funding, or salary from any organization that holds or has applied for patents relating to the content of the manuscript.

- They don't have any other financial or non-financial competing interests.

## Authors' contributions

All authors were responsible for the development of the study design. GV and MW have conceived the study. ASaA, PC, FB and FD have been involved in writing up, revising and optimising the study protocol. FD, MW, PC and FB coordinated the study in their respective university hospital center. ALD, SD and the clinical investigation center are responsible for inclusion of patients in the study and safety monitoring. PG and CL are involved in the national development of the study and the supervision of the work. All authors have read and corrected the draft versions and all authors contributed to and approved the final manuscript.

## Pre-publication history

The pre-publication history for this paper can be accessed here:

http://www.biomedcentral.com/1471-244X/11/1/prepub
